# Are mice a bad model for successive negative contrast?

**DOI:** 10.3758/s13420-025-00686-z

**Published:** 2025-10-07

**Authors:** Alan M. Daniel

**Affiliations:** Department of Health and Behavioral Sciences, Texas A&M University – San Antonio, One University Way, San Antonio, TX 78221 USA

**Keywords:** Incentive contrast, Reward downshift, Frustration effect, Negative contrast

## Abstract

Successive negative contrast (SNC) is a procedure in which animals trained with a large reward consume less of a subsequent smaller reward than animals always trained with the small reward. Studies of SNC in rats have emerged as an important tool in understanding the affective neuroscience of unexpected loss. Establishing a similar procedure in a murine model would allow access to a greater toolbox of neuroscience techniques (e.g., optogenetics, transgenics) that are more readily available in mice than rats. While the rat SNC literature has been thriving for decades, only a few studies report SNC effects in mice. This paper critically reviews the current literature on SNC in mice and presents a failure to replicate SNC using procedures commonly used in rats. Overall, the limited evidence available in mice and a lack of consistent findings suggest that mice may not be the most suitable model for studying the neurobiology of frustration, particularly when compared to the more established rat model.

## Introduction

*“For every problem in a given discipline of science, there exists a species or other entity or phenomenon ideal for its solution. Conversely, for every species or other entity or phenomenon, there exist important problems for the solution of which it is ideally suited.”* – E. O. Wilson, Letters to a Young Scientist

Reward values depend on both the absolute value of the reward and the organism’s previous experiences with other rewards. Animals that are first trained to expect a large reward then downshifted to a smaller reward will exhibit a disruption of behavior compared to a group that always received the smaller reward – a phenomenon known as successive negative contrast (SNC; Flaherty, 1996).

While this phenomenon has been widely observed across mammalian species, most of the studies regarding mechanisms of behavior, pharmacology, neuroanatomy, etc. have been conducted with rats serving as the gold standard for studying SNC. Notably, studies with rats have explored many subcortical circuits implicated in frustrative nonreward (see Hagen, Suarez, & Papini, 2025; Ortega et al., 2017). However, some important tools for neuroscience research are more widely available in mice (e.g., transgenic strains), and expanding SNC to a mouse model holds a lot of promise for advancing our understanding of reward comparisons. However, I contend that E. O. Wilson’s assertion that there is a species ideally suited to exploring any scientific problem implies that there are also species that are wholly unsuited to exploring that problem. In the case of SNC, I argue that while rats have supported a successful model for the neuroscience of surprising nonreward, mice are not tiny rats and likely fall into the latter category. Mice do not reliably exhibit SNC in the laboratory under the conditions that are necessary for a sufficiently advanced exploration of the phenomenon.

A limited number of studies have been reported in mice to date. Anecdotally, I am aware of at least three failed attempts to observe SNC in mice which have gone unpublished, all from different labs. The dearth of mouse studies in this area combined with anecdotal reports suggests there may be a file drawer problem in this area of study. If there are constraints on the conditions under which SNC is observable in mice, or if there are species-specific factors that make them unsuitable for SNC research, then researchers need to be aware of this to minimize failed efforts and wasted resources. Here, I critically review the literature on SNC in mice, and present a failure to observe SNC in mice using parameters commonly used in the rat paradigm. The data presented here cast doubt on the feasibility of directly translating SNC across rodent models.

### Successive negative contrast (SNC) procedures

One of the most well-studied versions of the SNC procedure measures consummatory behavior (cSNC). In the standard cSNC procedure, a group of rats are allowed 5 min free access to 32% sucrose for a 10-day preshift period, then allowed to drink 4% for an additional 5-day postshift period (Vogel, Mikulka, & Spear, 1968). If the downshifted group drinks less of the 4% sucrose compared to an unshifted control group that received only 4% throughout training, cSNC has been observed. The dependent variable is either cumulative number of licks or cumulative time spent licking during the trial (goal-tracking time). This can be referred to as the *standard procedure.* Except in unusual circumstances, both preshift and postshift phases are presented, with at least the critical postshift trials plotted to show the downshift and recovery over time. The key comparison that indicates SNC has been observed is a significant difference between shifted and unshifted groups during a portion of the postshift phase, when both groups have access to the smaller reward.

Another well-known but less widely used SNC procedure measures instrumental behavior (iSNC) following a quantitative or qualitative shift in reward (e.g., five food pellets). In a typical iSNC experiment, rats are trained to traverse a runway for a preferred reward for several days, followed by a postshift period where they traverse the runway and find a less preferred reward (e.g., one food pellet). If the latency of the downshifted rats is higher compared to a group of rats that always had the lesser reward, iSNC is observed. One notable element of the iSNC procedure is that the behavior measured is anticipatory, such that behavior measured on the first downshift trial is actually occurring before the experience with the downshifted reward. Therefore, any difference in latency would be expected to appear on the following trial at the earliest. In the seminal investigation of iSNC in a runway, the difference in speed to traverse the runway took time to emerge, peaking around four to six trials after the downshift (Crespi, 1942).

The two procedures differ in important ways, and a review of the similarity and distinction is beyond the scope of the present paper, but cSNC and iSNC can be considered overlapping but discriminable phenomena (Flaherty, 1982; Flaherty, 1996; Sastre, Lin, & Reilly, 2005). Arguably, the cSNC procedure with rats has been more useful for studying the neuroscience of reward downshifts due to several key features. First, the standard cSNC procedure is short, requiring only 5 min of access to sucrose per day. This allows study of treatments (e.g., drugs, behavioral pretreatments) that are of short duration or have transient or multiphasic effects. Because of the long recovery period, not only can the initial response to the downshift be explored, but also differences in rates of recovery. Differences in recovery rates have been proposed to a key indicator of vulnerability or resilience following a loss event (Papini, Wood, Daniel, & Norris, 2006). Pharmacological studies using the standard procedure have allowed the double dissociation of mechanisms active on the first and second downshift trials, attributed to *primary frustration* resulting from the initial emotional reaction to the downshift, followed by acquisition of a conditioned *secondary frustration* that disrupts future behavior (Papini et al., 2006).

Other behavioral outcomes are possible besides the observation of SNC (for review, see Papini, 2003). For example, downshifted animals may rapidly adjust to the new reward, responding as though there were no prior experiences with reward. This pattern of results only requires the animal to track the *magnitude of reward* without regard to prior experience (MR explanation). Alternatively, downshifted animals may gradually adjust to the new reward over time, or not adjust at all and continue to respond as though the large reward were still present. This pattern of results can be explained through a simple *strengthening-weakening rule* (SR explanation). SNC is only evident when the change in behavior cannot be accounted for by MR or SR explanations.

### Published studies reporting SNC in mice

The current literature does not demonstrate cSNC with mice as a clear corollary to the phenomenon observed in rats. In many of the published studies, critical information is excluded from the manuscripts, such as the trial duration, preshift patterns of behavior, or drug injection times. One frequent mistake is to consider a significant statistical interaction between preshift and postshift performance in the shifted and unshifted groups to indicate SNC. Any of the previously described patterns of behavior (MR, SR, SNC) could produce a significant statistical interaction. Instead, the preshift and postshift should be analyzed separately, or pairwise comparisons in the postshift phase can be utilized to indicate that the postshift behavior differed between groups, without contamination by preshift performance in the analysis.

Mustaca, Bentosela, and Papini (2000) were the first to report cSNC in mice. They used a substantially modified procedure in which two bottles were left in the home cage for extended periods of 1–3 h. This was necessary because the overall low consumption of fluids by mice meant that the standard 5-min paradigm was insufficient to discriminate consumption between groups. There were ten preshift trials and two postshift trials. The data revealed that mice preferred 32% sucrose to 4%, and that downshifted mice drank less of the 4% sucrose compared to unshifted controls. The results were more transient than what is typically observed in rats, lasting a single trial rather than showing gradual recovery of consummatory behavior over a 3- to 5-day period. The cSNC was reduced by administration of diazepam, a benzodiazepine anxiolytic, which suggests at least some common mechanisms with rat cSNC. The authors did not specify when the drug was administered relative to training. While this procedure was successful at producing cSNC, it precludes some types of investigation that are possible with rats. The effects were small and transient. For example, the unshifted group’s behavior is near the floor, so there is no room to look for treatments that enhance cSNC. It takes longer than the standard procedure, which is less practical in terms of throughput, but also prevents investigation of acute aftereffects of the downshift (e.g., hypoalgesia following the downshift in rats; Mustaca & Papini 2005). The time constraint makes it difficult to discern the time-course effects of various treatments, since many treatments have transient effects that may diminish before the end of the trial (e.g., formalin-induced pain enhances cSNC in rats; Ortega et al. 2011). This procedure is not sensitive enough to allow for the dissociation between the first and second downshift trials, nor does it allow for the study of the recovery period. Finally, there are potential issues with the use of diazepam in particular to validate this procedure mechanistically, since other studies with diazepam in mice have reported no anxiolytic effects, only sedative effects at high doses similar to those used in this study (Pádua-Reis, Nôga, Tort, & Blunder, 2021). Other studies have shown that the BALB/c mice used in this experiment are less sensitive to diazepam than other strains (Lepicard et al., 2000), but the nuance surrounding anxiolytic effects of diazepam in mice needs further exploration.

Austen, Strickland, and Sanderson (2016) explored how shifts in reward magnitude impact sucrose palatability. They measured cumulative lick frequency, volume consumed, and also lick cluster size in mice exposed to either a 32–4% downshift or a 4–4% unshifted control group. Trials were 15 min long, 5 min context exposure followed by 10 min of sucrose access. There were eight pre-shift sessions followed by a single test session. Pre-shift data were not presented, but in the post-shift session there were no differences in licks or volume consumed. The unshifted mice showed an elevated lick cluster size relative to the shifted group, but this was only evident during the first 2 min of the 10-min session, after which their lick cluster size reduced to match the downshifted group. The reported effect is far more transient than the effects typically observed with rats, and the difference is driven by the changing behavior of the unshifted group rather than the control group. If it is the case that the unshifted group typically begins the session with larger clusters and then reduces over time, then smaller clusters in the shifted group can fairly be attributed to SNC. This is counter to the argument by these and other authors (Austen & Sanderson, 2016; Naneix, Peters, & McCutcheon, 2020) that palatability determines lick cluster size and is relatively insensitive to the effects of satiation, whereas rodents decrease the number of bouts (rather than the number of licks per bout) to reduce intake as they become satiated. An alternative explanation is that prior training with 32% sucrose results in more stable cluster size across the trial than 4%, and the same pattern of results would be evident before the downshift. Without the preshift data for both groups to rule out MR or SR explanations, this cannot be confidently considered a demonstration of SNC in mice. Austen and Sanderson (2016) used a within-subjects design and observed the same transient difference between shifted and unshifted groups only during the first 2 min and driven by the reduction of lick cluster size in the unshifted context. While the within-subject design adds plausibility, alternative explanations to SNC cannot be ruled out.

Casaril et al. (2021) reported cSNC in mice, evidenced by lower volume consumed, lower number of licks, and smaller bout durations. They were primarily concerned with whether immune response triggered by lipopolysaccharide would influence SNC. While there was no effect of immune treatment, their observation of cSNC warrants attention. Preshift training involved seven daily sessions of unreported duration. The last 3 days of this phase were averaged together to establish baseline performance, and this was compared to a single downshift session. One experiment tested 12–2% versus 2–2%, another 12–4% versus 4–4%, and a third 12–1% versus 4–1%. In all of these experiments there was a reduction in consummatory behavior for downshifted animals compared to their preshift performance with the larger solution, which the authors interpret as cSNC. This observation only shows that the mice distinguished between the two solutions. The critical comparison that must be made to establish cSNC is between the postshift performance of the downshifted group and the unshifted group, which the authors never made. Visual inspection of the data suggests that in reality the 12–2% group drank more than the 2–2% group, indicating that there was in reality no cSNC in any of these experiments. The authors also conducted tests of iSNC with both quantitative and qualitative shifts, but the results are similarly obfuscated because the critical comparisons are missing.

Spangenberg and Wichman (2018) report iSNC in mice following a qualitative downshift from a neutral food pellet to a preferred hazelnut cream reward. The dependent variable was latency to traverse the runway for food. Significant differences were found, but in the opposite direction to what would indicate iSNC. Instead, the mice trained with the large reward continued to run faster for the first 2 days after the shift. It is important to remember that iSNC involves anticipatory behavior, which means behavior measured on the first downshift trial is actually occurring before the experience with the downshifted reward. In any case, there was no iSNC observed, and if anything, the results are better suited to the SR or MR explanations. The authors suggest that the decrease in the downshifted group after the downshift is suggestive of SNC, but the same result would be expected under MR and SR explanations, so this cannot be considered a demonstration of iSNC.

Clarkson, Leach, Flecknell, and Rowe (2020) report cSNC in mice in a procedure that is closest to the standard procedure to date. In the preshift, mice received either 32% or 4% sucrose for ten daily 15-min trials. Half of the mice were then shifted to the other solution for eight more sessions to look for positive and negative contrast effects. The mice tended to drink more of the 4% than the 32% sucrose during the preshift, so SNC effects were not explored for volume consumed. They did find reduced lick cluster sizes for downshifted mice compared to controls during the postshift, an apparent cSNC effect. In contrast to Austen, Strickland, and Sanderson (2016), the change could not be attributed to differences in the unshifted controls, and the inclusion of the preshift data eliminated MR and SR explanations. There was one problem, however: rather than analyzing the raw data across the eight postshift sessions, each mouse’s postshift behavior was averaged across four of the trials to create early and late postshift “phases.” According to the authors, this was necessary because mice drink small amounts which increases variability in lick cluster sizes across trials. While this is undoubtedly true, this practice is statistically dubious and should be resolved methodologically rather than through data transformation. The statistical test used computes a signal:noise ratio, and the variability in lick clusters is what determines the noise. Averaging out the noise by combining multiple measurements from the same individual artificially reduces variability between subjects, increasing the chances of type I error (Drummond & Vowler, 2012). There is also no justification for why there would be two “postshift phases,” as opposed to another method such as presenting a cumulative count of postshift licks overall. As such, the data from this study cannot be convincingly considered a demonstration of cSNC in mice. Even if one decides that averaged postshift data are acceptable, pooling data across sessions is not necessary with the standard procedure in rats. The necessity to transform data supports the idea that mice are too inconsistent under these conditions to be used as a standard model. The author’s experiences with high individual variability across trials and poor consumption of the 32% sucrose mirror my own experiences and anecdotal reports from multiple labs.

Together, these studies do not put forward a convincing murine analogue of SNC that is robustly observed in rats. At a minimum, such a model must show a difference in postshift between downshifted and unshifted mice which cannot be explained by previous patterns of behavior in the preshift. Second, the murine model would need to be validated as sharing mechanisms with the phenomenon as observed in rats. The ideal procedure would allow investigation of acute treatments, primary and secondary frustration, and differences in recovery rates in the same way as the rat preparation. A high-throughput murine model that allows investigation of these features would have considerable value to neuroscience.

To investigate the feasibility of using the standard procedure with mice, I conducted a study using the best principles described above. A straightforward demonstration of mouse SNC should be evident with the correct parameters. Here, I present a failure to detect SNC with a variety of sucrose solutions under parameters that have been shown to provide useful data in rats.

## Method

### Subjects

A total of 64 C57BL/6 male mice (Jackson Labs, https://www.jax.org/strain/000664) were housed in standard large plastic mouse tubs with free access to food and water until aged 90 days. At 90 days, each mouse was separated into individual housing and placed on food restriction, with a target of 85% ad libitum weight based upon 3 days of free-feeding weights. At no time did any mouse drop below 80% of their ad libitum weight. All animals were housed and trained under an approved protocol from the Institutional Animal Care and Use Committee at Texas A&M University – San Antonio.

### Apparatus

Testing chambers were custom-built three-dimensional printed modular mouse test chambers (Mazziotti et al., 2020) measuring 16 × 16 × 16 cm, with a custom front wall installed with an oval-shaped hole that allowed for the insertion of a sipper tube. Sipper tubes were affixed to 10-ml plastic syringes containing the test solutions, which were mounted to a geared plate. A stepper motor inserted the sipper tube precisely into the apparatus flush with the wall. Each test chamber had its own Raspberry Pi computer attached which automatically controlled insertion and retraction of the sipper tube, as well as goal-tracking. Goal-tracking was collected via a capacitive sensor attached to each sipper tube which detected licks. Lick data were saved to a cloud server following each trial.

### Sucrose solutions

A downshift of 32% to 4% sucrose (W/W) is typical of the solutions used for SNC studies in rats. However, pilot data I collected suggested that 32% sucrose was not palatable to the mice, as many mice failed to acquire preshift drinking behavior. For this reason, I opted to include sucrose solutions of 32%, 16%, 4%, and 2% to capture a wider range of reward values, all of which have successfully produced SNC in rats.

### Procedure

Prior to training, mice were divided into four groups, receiving 32%, 16%, 4%, or 2% in the preshift phase. Prior to the first downshift trial, the 32% and 16% mice were randomly matched according to their preshift performance to be subdivided into groups to be tested with either 4% or 2%. The final six groups tested (pre-post) were 32–4%, 16–4%, 32–2%, and 16–2% downshifted, with groups 4–4% and 2–2% as unshifted controls for comparison. Mice were trained in a single trial per day lasting 5 min. Training continued for ten pre-shift and ten post-shift trials. There were four testing boxes, so animals were trained in squads of four at a time, always in the same squads and each mouse always in the same testing chamber. The order of squads was randomized each day. Chambers were wiped with a damp cloth in a random order after each mouse to distribute odors evenly. Mice were placed in the boxes and the trial was started via computer. Following a minimum 30-s pre-trial interval, the sipper tube was inserted into the box and the mouse was allowed to drink for 5 min following the first contact with the sipper tube. When the trial ended, mice were repatriated to their home cages, and after all mice completed training, were fed standard rodent chow.

## Results

Cumulative goal-tracking times (s) were observed for mice drinking sucrose solutions for ten pre-shift and ten post-shift trials. All data analyses were conducted using JASP statistical software version 0.19.1 using type III sum of squares. Data were lost for eight of the 1,280 data points; missing data were imputed using the multivariate imputation by chained equations (MICE) package in R statistical software. Pre-shift and post-shift imputations were completed separately. The pre-shift and post-shift phases were compared in two separate analyses.

### Pre-shift

A 4 × 10 (Solution × Trial) mixed-methods ANOVA was conducted to compare baseline sucrose consumption. Assumption testing indicated that the sphericity assumption was violated, so a Huynh-Feldt correction was applied. There was a medium significant main effect of Trial, *F*(6.93, 415.94) = 9.94, p < 0.01, ω^2^ = 0.07, indicating that mice learned to drink more sucrose as trials progressed. There was also a small but significant Solution × Trial interaction, *F*(20.78, 415.94) = 2.83, p < 0.01, ω^2^ = 0.02, indicating that the mice learned to drink 16% and 32% faster than 2% and 4% solutions. There was no main effect of Solution, indicating that overall mice drank similar amounts of all solutions, *F*(3, 60) = 2.20, p = 0.10, ω^2^ = 0.01.

### Post-shift

**Fig. 1:**
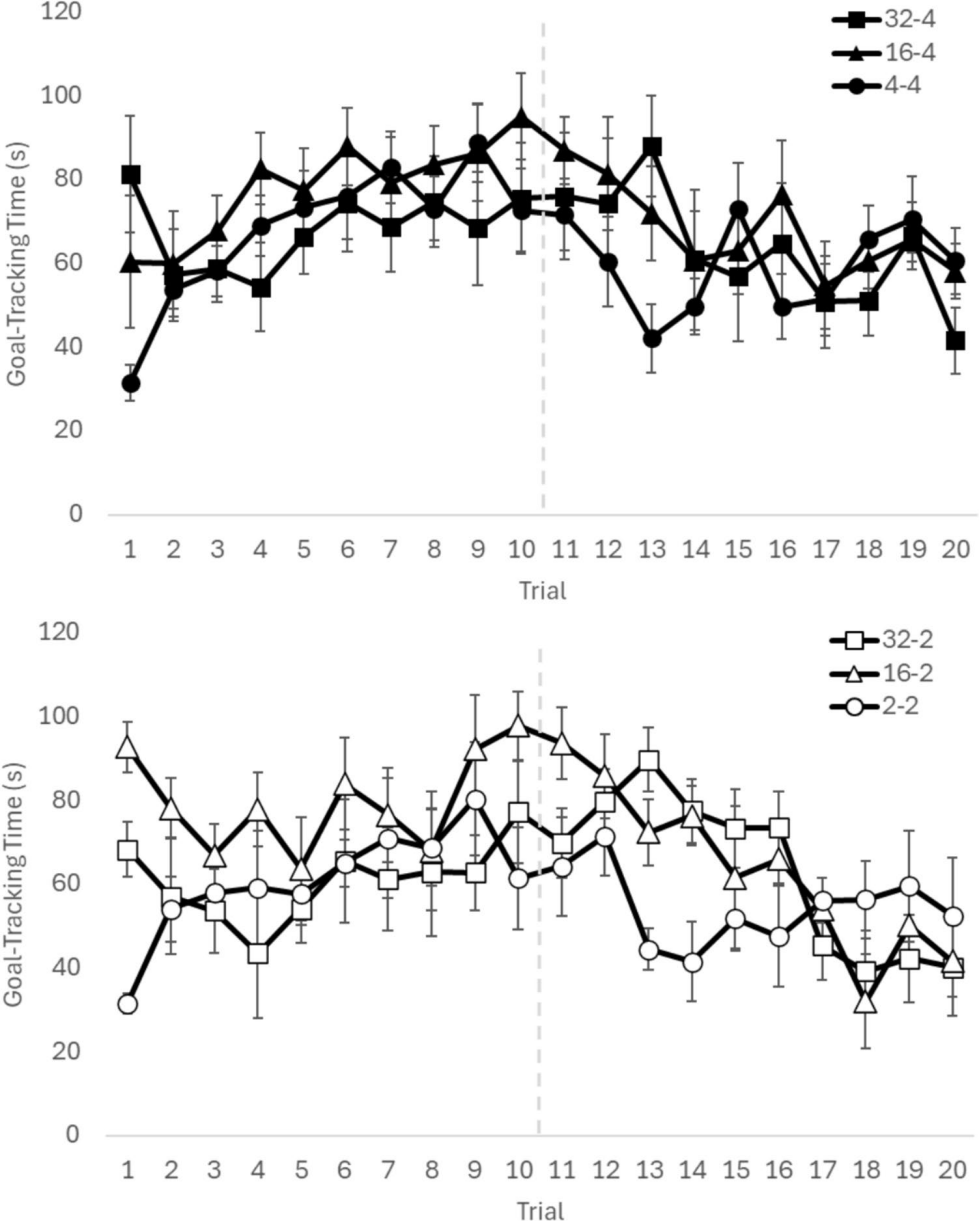
Goal-tracking time in seconds for each group for each trial. Error bars are +/- SEM

Any evidence of SNC will be observed in the post-shift phase, with the critical comparisons between downshifted groups and their respective unshifted group drinking the same solution (i.e., 32–4% or 16–4% compared to 4–4% and 32–2% or 16–2% compared to 2–2%; see Fig. 1). A 6 × 10 (Group × Trial) mixed-methods ANOVA was conducted to compare sucrose consumption across groups in the post-shift phase. Assumption testing indicated that the sphericity assumption was violated, so a Huynh-Feldt correction was applied. There was a small significant main effect of Trial, *F*(7.03, 407.08) = 7.05, p < 0.01, ω^2^ = 0.05, indicating a general trend to drink less as post-shift trials progressed. There was no main effect of Group, *F*(5,58) = 1.39, p = 0.24, ω^2^ > 0.01. There was a small but significant Group × Trial interaction, *F*(35.13, 407.08) = 2.08, p < 0.01, ω^2^ = 0.01, which could be indicative of contrast if the downshifted groups drank less than the control group. To explore this possibility, one-way ANOVAs were conducted for each post-shift trial with Group as the independent variable. Only trials 13 and 14 showed significant differences with small effect sizes, and pairwise comparisons of the groups indicated that the downshifted groups were drinking more than the unshifted groups, not less, thus SNC was not evident.

## Discussion

Despite a thriving literature on consummatory successive negative contrast in rats using a well-established standard procedure, only a handful of reports in the published literature demonstrate a similar effect with mice. Of these studies, the methods are not standardized and most have statistical issues that cast doubt upon the interpretation that mice showed cSNC. The present study addressed these issues and showed a clear failure of cSNC using sucrose solutions in mice, suggesting a murine model may not be the best use of resources for answering questions about surprising reward loss. If anything, the data presented here support an SR explanation of mouse responses to downshift, with training with larger rewards resulting in persistence following the downshift consistent with the graphs shown in Casaril et al. (2021), which is commonly reported in non-mammalian vertebrates (Papini, 2003).

There are several possibilities regarding why mice may not be as sensitive to reward downshifts. One possibility is that the cSNC observed readily in rats is a species-specific phenomenon. Given the widespread observation of incentive contrast across mammalian taxa, this seems unlikely, but many of the studies in species other than rats do not use downshifts in sucrose solutions as the shifted rewards. While there are reports of SNC in humans, there is no gold standard procedure established and it is not clear how well the rat literature directly translates to the human observations of SNC. It may be that the rat model is the most well suited to this field of research.

A second possibility is that observation of cSNC is highly dependent on body size. Mice may become satiated earlier, or have lower overall motivation to consume sucrose compared to rats. In rat studies, it is common to use larger strains or use exclusively male subjects even though females exhibit cSNC because intake appears to be correlated with body size; though a systematic exploration of sex and strain differences in rat cSNC is yet to be done. For mice, sucrose response is lower than rats, with their consumption curves peaking at concentrations of around 10% for many strains (Lewis et al., 2005). In order to show cSNC there must be a high enough disparity between solutions such that it generates the effect, but also enough consumption of the lower solution to show a difference, i.e., avoid floor effects. The interaction of body size and sucrose response means that it is difficult to find a combination of sucrose solutions to achieve this in mice. If the large solution is too large, they will not consume it. If the solution is too small, they will not consume it. The range of solutions available in the “Goldilocks zone” may be too narrow in mice for studying cSNC.

Body size alone may be insufficient to explain the difference in cSNC in rats and mice, since infant rats have been shown to exhibit cSNC (Stanton, Lobaugh, & Amsel, 1984) and SNC-like taste reactivity patterns (Suarez et al, 2017), as well as positive contrast (Avellaneda, Serafini, & Kamenetzky, 2022). I suggest that across species lines, body size could be a correlate of metabolic rate and energy demands. In the case of cSNC, it could be that abandoning a diminishing food source too early is more costly for mice than for rats, and thus a slower onset of frustration is optimal in the long run. In terms of optimal foraging, the relatively low energy demands of mice compared to larger rats means that the point at which a foraging patch becomes unprofitable comes much later for mice than rats (Bedoya-Perez et al., 2013; Cozzoli et al., 2018). Mice can therefore exploit lower quality patches for longer and still maintain their high metabolic needs. The data presented here may actually be consistent with this idea. On trials 18–20, the 32–2% group responded numerically lower than the 2–2% group. This could be interpreted in terms of an emerging cSNC effect after extensive experience with the lesser reward. If training were to be continued for more sessions perhaps this difference would be measurable and more pronounced; or if trial durations were extended then the mice may get more experience with the rewards more quickly. There are other situations where mice have exhibited slower-to-emerge behavior relative to rats, such as in adaptive decision-making (Jaramillo & Zador, 2014) and fear extinction (Bisby et al., 2021). While it is tempting to characterize mice as small rats who are not as smart and need more time to learn, a more nuanced view is that mice adopt different foraging strategies shaped by their natural history. For example, Jones et al. (2017) demonstrated that rats will readily learn to *withhold action* (no-go) to avoid punishment and to receive rewards in a shuttle box but struggle to learn to *take an action* (go) for the same outcomes. Mice, on the other hand, are the opposite, and learn the “go” tasks more readily than the “no-go” tasks. Training the mice under the contingency in which the rats excel would make their performance worse, whereas the conditions under which mice outperform the rats might be missed.

If this experiment were to be repeated, a longer trial duration and extending the number of post-shift sessions would be a good starting point. If it is true that mice require more extensive reward experience and have a slow-to-emerge cSNC, then that is an interesting difference relative to the immediate reaction of rats. If this is the case, it is an interesting finding in its own right, but if the goal is a rapid throughput animal model, then adding more trials of longer length works against that goal.

Mice may be better suited to other kinds of species-specific reward downshift effects besides sucrose solutions. For example, perhaps they would be more sensitive to protein, lipid, or starch concentrations. Alternatively, sucrose rewards can be devalued in other ways, such as adding quinine instead of a shift to a lower concentration (Flaherty & Rowan, 1989). An observation of this kind would require systematic testing of different solutions, followed by validation of any observed cSNC effect to establish common mechanisms (e.g., common neurochemical systems, common neural circuitry).

There are likely to be strain differences in mice for cSNC effects. The present study used C57BL/6 mice due to their popularity, but it is possible that another strain may be more sensitive to reward downshifts. While strain differences could be considered a feature rather than a deficit, determining which strain compares best to what we know from rat procedures is an empirical task. Validating different strains with different manipulations is tedious and not very interesting. If someone were to undertake such a project, would it be sufficiently valuable or novel for journals to even publish it? Is it worth someone’s time, when it may turn out that no strain produces an adequate corollary? It does not seem like the best use of limited resources, unless it can be added on to another research question that happens to be suitable, such as adding a downshift session following extended sucrose consumption in a study of metabolism. If the goal is to test existing strains of transgenic mice, then there is no guarantee that the strains used to develop the mutant mouse will exhibit SNC, requiring extensive testing of wildtype phenotypes from the same strain and hoping there is a difference.

Handling method may also play a role in mouse anxiety-related behaviors. Clarkson et al. (2018) observed decreased sucrose reward responsiveness for tail-handled mice compared to tunnel-handled mice, and greater anxiety-related behaviors in an open field and elevated plus maze. In the present study, mice were handled via tails, which may have artificially suppressed the higher reward values. Other studies have shown that the C57BL/6 strain used here may not be as sensitive to the tail handling, and while there are effects on measures of anxiety based on handling type, it does not always show up in terms of physiological measures of chronic stress (Novak et al., 2022). In any case, handling method may also be a relevant variable to keep in mind during any anxiety-related behavioral testing, with tunnel handling as a better option.

There is no doubt that expanding the tools to understand SNC is a valuable goal. When establishing a new standard procedure for observation of SNC, it is critical that observation of SNC is clear and unambiguous with a strong effect size. While SNC tends to be an ephemeral phenomenon, a barely detectable effect that lasts only a single trial is unlikely to lead to a robust literature. Additionally, there needs to be enough behavior generated in the task to enhance or reduce the effect using manipulations, avoiding floor and ceiling effects. Ideally, cSNC in a new procedure would be able to be validated as homologous with the rat SNC literature in terms of neurocircuitry or pharmacology. Given the current state of the literature, mice do not appear to be best suited to exploring SNC behavior.

A promising alternative to the SNC procedure has recently emerged in the literature, the alternate poking reward omission (APRO) procedure (Naik et al., 2024; Papini et al., 2024). In this procedure, water-restricted mice are trained in a runway with goal areas at each end. The mouse is rewarded with water for alternating pokes on either end of the runway, and learns to shuttle between the two goal areas. Daily trials are 15 mins long. For 3 days of training they are continuously reinforced, and on the fourth and fifth days, the reward schedule is shifted to partial reinforcement at 50% and 20%, respectively. Compared to continuously reinforced controls, shifting to reward omissions results in increased running speeds and reward port visits. Evidence of frustration in this task can be seen from increased locomotor activity and aggressive behaviors in tests immediately following APRO compared to continuously reinforced controls. APRO is promising because it is a simple and relatively short-term procedure with brief daily trials, which allows exploration of things like drug administration that requires an acute frustration induction. However, it is not a perfect corollary to cSNC procedures. All of the behavior is anticipatory rather than consummatory, so it shares many of the limitations of iSNC procedures described above. Reward probabilities are manipulated instead of reward magnitudes, which may impact some research questions (e.g., ratio scaling of reward comparisons; Daniel, Ortega, & Papini, 2009; Papini & Pellegrini, 2006; Pellegrini & Papini, 2007). As a new procedure, further validation to establish its utility among procedures that elicit frustration is warranted.

## Data Availability

Data are publicly available via the Open Science Framework repository DOI: 10.17605/OSF.IO/DP7ZH
